# Lactate Metabolism in Breast Cancer Microenvironment: Contribution Focused on Associated Adipose Tissue and Obesity

**DOI:** 10.3390/ijms21249676

**Published:** 2020-12-18

**Authors:** Andjelika Kalezic, Mirjana Udicki, Biljana Srdic Galic, Marija Aleksic, Aleksandra Korac, Aleksandra Jankovic, Bato Korac

**Affiliations:** 1 Institute for Biological Research “Sinisa Stankovic”, National Institute of Republic of Serbia, University of Belgrade, 11000 Belgrade, Serbia; andjelika.kalezic@ibiss.bg.ac.rs (A.K.); aleksandra.jankovic@ibiss.bg.ac.rs (A.J.); 2 Faculty of Medicine, University of Novi Sad, 21000 Novi Sad, Serbia; mirjana.udicki@mf.uns.ac.rs (M.U.); biljana.srdic-galic@mf.uns.ac.rs (B.S.G.); 3 Faculty of Biology, University of Belgrade, 11000 Belgrade, Serbia; marija.aleksic@bio.bg.ac.rs (M.A.); aleksandra.korac@bio.bg.ac.rs (A.K.)

**Keywords:** lactate, lactate dehydrogenase, premenopausal breast cancer, cancer-associated adipose tissue, obesity

## Abstract

Metabolic reprogramming that favors high glycolytic flux with lactate production in normoxia is among cancer hallmarks. Lactate is an essential oncometabolite regulating cellular redox homeostasis, energy substrate partitioning, and intracellular signaling. Moreover, malignant phenotype’s chief characteristics are dependent on the interaction between cancer cells and their microenvironment. In breast cancer, mammary adipocytes represent an essential cellular component of the tumor milieu. We analyzed lactate concentration, lactate dehydrogenase (LDH) activity, and isozyme pattern, and LDHA/LDHB protein expression and tissue localization in paired biopsies of breast cancer tissue and cancer-associated adipose tissue in normal-weight and overweight/obese premenopausal women, compared to benign breast tumor tissue and adipose tissue in normal-weight and overweight/obese premenopausal women. We show that higher lactate concentration in cancer tissue is concomitant with a shift in isozyme pattern towards the “muscle-type” LDH and corresponding LDHA and LDHB protein expression changes. In contrast, significantly higher LDH activity in cancer-associated adipose tissue seems to be directed towards lactate oxidation. Moreover, localization patterns of LDH isoforms varied substantially across different areas of breast cancer tissue. Invasive front of the tumor showed cell-specific protein localization of LDHA in breast cancer cells and LDHB in cancer-associated adipocytes. The results suggest a specific, lactate-centric relationship between cancer tissue and cancer-associated adipose tissue and indicate how cancer-adipose tissue cross-talk may be influenced by obesity in premenopausal women.

## 1. Introduction

Metabolic reprogramming is one of the hallmarks of breast cancer that serves to sustain the malignant phenotype in response to the selective pressures imposed by the tumor microenvironment. It enables progression [1], metastasis [2], and resistance to conventional therapies [3]. A critical feature of adaptive cancer metabolism is an increased glycolysis rate with lactate production in normoxia, also known as the Warburg effect [4,5]. Lactate is an end product of glycolysis produced in a reaction catalyzed by lactate dehydrogenase (LDH). LDH is a homo/heterotetramer that consists of a combination of four LDHA (EC:1.1.1.27) or LDHB (EC:1.1.1.27) subunits, forming five distinct isozymes with tissue-specific distributions. LDHA homotetramer, also known as the “muscle-type” LDH or LDH5, preferably converts pyruvate to lactate and NAD+ and is a predominant isozyme expressed in muscle. In contrast, LDHB homotetramer, also called the “heart-type” LDH or LDH1, mostly converts lactate to pyruvate and NADH and is a predominant isozyme expressed in the heart. Most tissues, including cancers, express balanced, heterotetrameric forms of LDH (LDH2, LDH3, and LDH4) as well as combinations of several isozymes [6]. Recent studies revealed that lactate is an essential oncometabolite that enables continuous glycolysis, serves as an energy fuel for oxidative metabolism, and represents a valuable precursor for biosynthesis [7]. Moreover, lactate is indispensable in the regulation of gene expression, energy substrate partitioning, and cellular redox homeostasis [8]. Accordingly, many studies showed altered lactate metabolism in breast cancer. Namely, high lactate concentration [9,10] and increased LDH activity [11] were reported as a consequence of overexpression of LDHA enzyme subunit [12], underexpression of LDHB enzyme subunit [13], and an overall shift towards the expression of “muscle-type” LDH [14,15]. Besides, all of the above characteristics are related to cancer stemness, survival, aggressiveness, metastatic potential, and patient survival rates making lactate metabolism a potentially useful biomarker in breast cancer diagnosis, prediction, and therapy [12,16].

Furthermore, breast cancer should be considered a complex pseudo organ since the pivotal characteristics of cancer cells are deeply dependent on the interaction with their microenvironment [17]. A plethora of stromal components, including fibroblasts, vascular endothelial cells, mast cells, and immune cells, as well as components of the extracellular matrix, are involved in shaping a complex cancer-promoting niche [18]. Recently, mammary adipocytes have been recognized as an important cellular component of the tumor microenvironment that plays a key role in breast cancer development. These cancer-associated adipocytes promote cancer progression through paracrine action of growth factors, adipokines, and proinflammatory cytokines [19]. Emerging evidence points to the metabolic cross-talk as one of the integral aspects of cancer-adipose tissue two-way communication. As the body’s main sites of glucose and lipid homeostasis, adipocytes can provide substantial metabolic support to proliferating cancer cells through the supply of substrates such as ketone bodies, glycerol, and fatty acids [20,21]. Moreover, carbohydrate and lipid metabolism in adipose tissue are intertwined with complex lactate metabolism [22,23]. Here, lactate can play a pivotal role in facilitating metabolic cooperation between cancer cells and adipocytes by serving as an intercellular messenger [24], a mediator of mutual metabolic reprogramming [25], and a substrate for oxidative metabolism and biosynthesis [26]. Such mechanisms of cooperation can particularly be important in obesity, where dysfunction of resistant adipose tissue is known to enhance the detrimental communication between cancer cells and adipocytes, thus promoting cancer aggressiveness [27]. However, there is a lack of data on in vivo depot-specific lactate metabolism of mammary adipose tissue, and the roles of lactate in cancer-adipose tissue cross-talk remain mostly unknown.

This study aimed to reveal changes to the lactate metabolism in breast cancer and its relationship with obesity in premenopausal women. Unlike in postmenopausal women, where there is a strong relationship between obesity and breast cancer incidence, the severity of the disease progression, and outcome, this relationship in premenopausal women remains controversial, with underlying mechanisms largely elusive [28]. Here, we set out to examine the fundamental breast cancer and adipose tissue metabolism in relation to obesity since mechanisms that were previously shown in postmenopausal women such as estrogen and progesterone levels, insulin resistance and hyperinsulinemia, adipokine levels, and adipose tissue inflammation [29] do not seem to provide sufficient explanation for the differences in disease outcomes between normal-weight and obese premenopausal women. A particular focus was given to basal lactate metabolism in mammary adipose tissue and its association with malignancy and obesity. To this end, we examined lactate concentration, LDH activity, and isozyme profile, as well as protein expression and tissue localization of LDHA and LDHB in paired biopsies of malignant tumor tissue and tumor-associated adipose tissue from normal-weight and overweight/obese women with breast cancer. Paired biopsies of tumor tissue and tumor-associated adipose tissue from normal-weight and overweight/obese women with non-malignant (benign) breast changes were used as controls.

## 2. Results

### 2.1. Lactate Concentration in Malignant Tumor Tissue of Premenopausal Women with Breast Cancer Reveals Obesity-Related Differences

**Figure 1:**
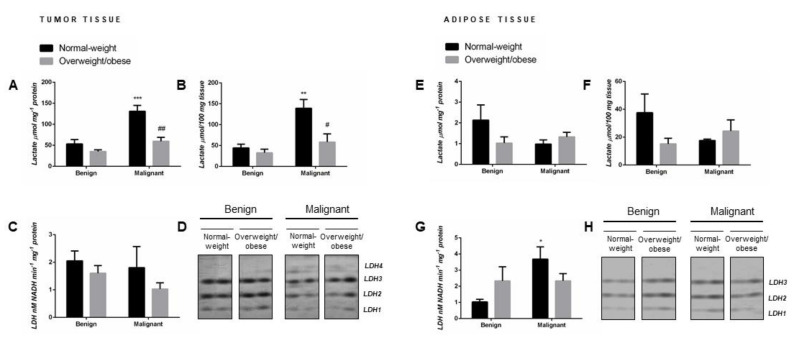
Lactate concentration per mg of protein (**A**,**E**) and per 100 mg of wet tissue mass (**B**,**F**), total lactate dehydrogenase (LDH) activity (**C**,**G**), and LDH isozyme profile (**D**,**H**—two representative band images cut from the same gel are shown) in breast tumor tissue (left) and adipose tissue (right) of normal-weight (black) and overweight/obese (gray) women. Lactate concentration and LDH enzyme activity are expressed in absolute units. Bars represent the mean ± S.E.M. * Compared to respective benign counterpart, * *p* < 0.05, ** *p* < 0.01, *** *p* < 0.001; # compared to normal-weight malignant counterpart, # *p* < 0.05, ## *p* < 0.01.

Malignant tumor tissue of normal-weight women showed significantly higher lactate concentration (µmol mg-1 protein) compared to their benign counterparts. Interestingly, in malignant tumor tissue of overweight/obese women, lactate concentration did not differ in comparison to benign tumor tissue of overweight/obese women but was significantly lower than in malignant tumor tissue of normal-weight women (Figure 1A). No malignancy- or obesity-related differences in tissue lactate concentration were found in tumor-associated adipose tissue (Figure 1E). The same results were obtained when lactate content was expressed per 100 mg of wet tissue mass (Figure 1B,F).

### 2.2. Malignancy Stimulates Lactate Dehydrogenase Activity in Tumor-Associated Adipose Tissue of Normal-Weight Premenopausal Women

Although malignant tumor tissue showed significant obesity-related differences in tissue lactate concentration, no differences were found in total LDH activity (nM NADH min^−1^ mg^−1^ protein) in benign and malignant tumor tissue samples from normal-weight and overweight/obese women (Figure 1C). However, tumor-associated adipose tissue of normal-weight women with breast cancer exhibited significantly higher total LDH activity in comparison to adipose tissue of normal-weight women with benign tumors, while no differences were found in overweight/obese women (Figure 1G).

### 2.3. Malignancy Changes the Isozyme Pattern of Lactate Dehydrogenase in Tumor Tissue of Premenopausal Women

The same distribution pattern of three isozymes (LDH1, LDH2, and LDH3) was detected in benign tumor tissue of normal-weight and overweight/obese women. Regardless of obesity, there was a shift in isozyme distribution towards the “muscle-type” LDH in malignant tumor tissue: namely, a decrease in band intensity corresponding to isozymes LDH1 and LDH2 with the appearance of the band corresponding to isozyme LDH4 (Figure 1D). In tumor-associated adipose tissue, there were no changes in isozyme pattern (LDH1, LDH2, and LDH3) concerning obesity or malignancy. However, there was a change in band intensity for all three isozymes corresponding to the changes in total lactate dehydrogenase activity in tumor-associated adipose tissue (Figure 1H).

### 2.4. Protein Expression of LDHA and LDHB Monomers Matches the Profile of LDH Isozymes but not Total LDH Activity in Tumor Tissue of Premenopausal Women

**Figure 2:**
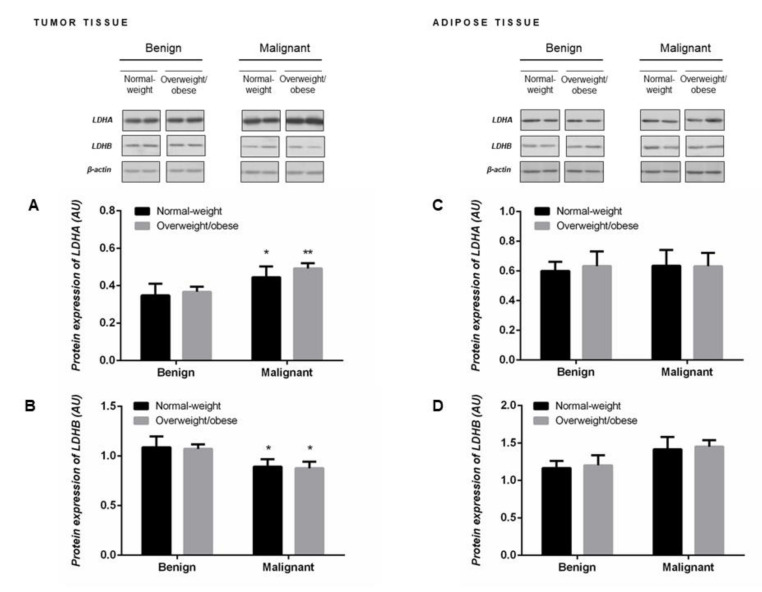
Protein expression of lactate dehydrogenase monomers LDHA (**A**,**C**) and LDHB (**B**,**D**) in breast tumor tissue (left) and adipose tissue (right) of normal-weight (black) and overweight/obese (gray) women. The protein content is expressed in arbitrary units (AU). Two representative band images cut from the same blot are shown; each band represents three pooled samples (*n* = 9, pooled by 3). Bars represent the mean ± S.E.M. * Compared to respective benign counterpart, * *p* < 0.05, ** *p* < 0.01.

Malignant tumor tissue of premenopausal women showed significantly higher protein expression of LDHA (Figure 2A) concomitant with lower protein expression of LDHB (Figure 2B). Protein expression of LDH monomers corresponded to the obtained profile of LDH isozymes, an increase in LDH4 isozyme expression at the expense of LDH1 and LDH2 isozymes (Figure 1D). No differences in the protein expression of LDHA and LDHB monomers were found in the tumor-associated adipose tissue between groups of normal-weight and overweight/obese women with benign and malignant tumors (Figure 2C,D).

### 2.5. Immunohistochemistry Reveals Opposing Trends in LDH Localization Patterns: LDHA in Cancer Cells and LDHB in Cancer-Associated Adipocytes

**Figure 3:**
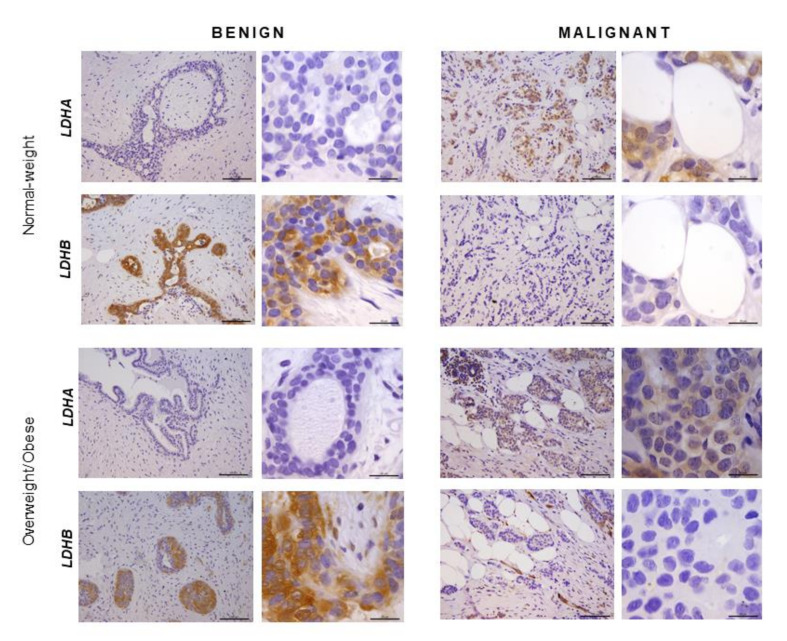
Immunohistochemistry and light microscopy showing LDHA and LDHB protein localization patterns in benign and malignant tumor tissue. LDHA (brown) protein localization in breast cancer cells in malignant tumor tissue and LDHB (brown) protein localization in breast epithelial cells in benign tumor tissue of normal-weight and overweight/obese premenopausal women. Scale bars: 100 μm and 20 μm.

**Figure 4:**
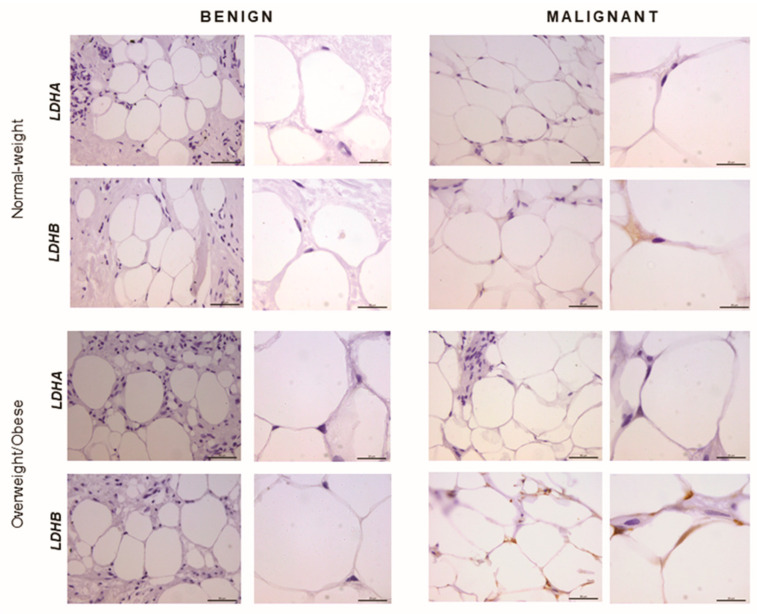
Immunohistochemistry and light microscopy showing LDHA and LDHB protein localization patterns in benign and malignant tumor-associated adipose tissue. Predominant LDHB (brown) protein localization in cancer-associated adipocytes, compared to adipocytes from benign breast tumors of normal-weight and overweight/obese premenopausal women. Scale bars: 50 μm and 20 μm.

**Figure 5:**
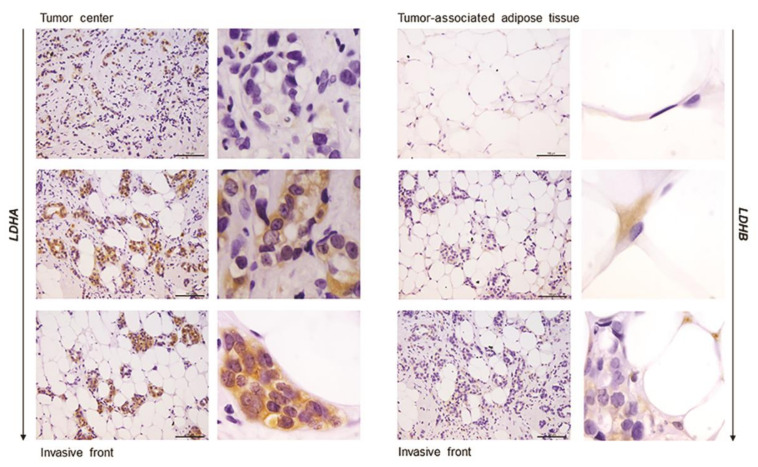
Immunohistochemistry and light microscopy showing a stark difference in cell-specific localization of LDH isoforms along the axis tumor center—tumor invasive front—tumor-associated adipose tissue. LDHA (brown) protein localization in breast cancer cells (left) and LDHB (brown) protein localization in cancer-associated adipocytes (right). Scale bars: 100 μm and 20 μm.

In order to confirm that the observed differences in LDHA and LDHB protein expression reflect changes specifically in breast epithelium and adipocytes, we further performed immunohistochemical analysis of LDHA and LDHB tissue protein localization. Indeed, LDHA was predominantly localized in breast cancer cells, in contrast to benign tumors where LDHB was predominantly localized in breast epithelium (Figure 3). Interestingly, in adipose tissue, LDHB was preferentially expressed in cancer-associated adipocytes in comparison to adipocytes found in benign breast tumors (Figure 4). Finally, we observed a stark difference in cell-specific protein localization of LDH isoforms, LDHA in breast cancer cells, and LDHB in cancer-associated adipocytes, while this phenomenon was more pronounced at the invasive front of the tumor, compared to the tumor center and distant tumor-associated adipose tissue, respectively (Figure 5).

## 3. Discussion

This study evaluated lactate metabolism in breast tumor tissue and tumor-associated adipose of normal-weight and overweight/obese premenopausal women. Cross-examination of benign and malignant tumor tissue biopsies revealed a significant shift in LDH isozyme expression towards the “muscle-type” LDH with corresponding changes in protein expression of LDHA (increased) and LDHB (decreased) in malignant tumor tissue, regardless of obesity. However, malignant tumor tissue of normal-weight women showed significantly higher tissue lactate concentration compared to overweight/obese women. Beyond cancer tissue, we report significant malignancy-related changes to the lactate metabolism in cancer-associated adipose tissue. That is a higher LDH activity, which could be attributed mainly to the “heart-type” LDH. Moreover, protein localization patterns of LDH isoforms varied substantially along the axis tumor center—invasive front—tumor-associated adipose tissue, where the invasive front showed a stark contrast in cell-specific protein localization of LDHA in breast cancer cells and LDHB in cancer-associated adipocytes. The results indicate a specific, lactate-dependent relationship between cancer tissue and cancer-associated adipose tissue in premenopausal breast cancer and suggest how cancer-adipose tissue cross-talk may be influenced by obesity.

Our results on normal-weight premenopausal women support and extend previous findings for breast cancer lactate metabolism. We showed significantly higher LDHA and lower LDHB protein expression in malignant tumor tissue than benign tumor tissue, which is a typical signature of luminal type A invasive ductal carcinoma [13,30]. Such expression profile supports the observed shift towards the “muscle-type” LDH in malignant tumors, evident as a higher prevalence of LDH3 and LDH4 and lower prevalence of LDH1 and LDH2 isozymes, which is mostly in accordance with available data for human breast cancer samples and corresponding breast cancer cell lines [14]. Considering the metabolic diversity of breast cancers [31] and metabolic plasticity of breast cancer cells [32,33], we proceeded to analyze tissue LDH protein expression and localization. Immunohistochemistry confirmed that LDHA was primarily localized in breast cancer cells in contrast to LDHB, which was primarily localized in breast epithelial cells within benign breast tumors. Several in vitro studies showed that overexpression of LDHA in cancer cells leads to increased proliferation and invasion as part of the epithelial-mesenchymal transition [34,35] as well as resistance to apoptosis and protective autophagy in tamoxifen resistance [36]. Our results support this view, where high LDHA protein expression is a metabolic prerequisite for highly-active, highly-anabolic, rapidly-proliferating, and migrating cells, such as those found at the invasive front of the tumor.

Recognizing that chief characteristics of the malignant phenotype are heavily dependent on the interaction between cancer cells and their microenvironment, we next analyzed one of the essential cellular components of the breast cancer milieu—the mammary adipose tissue. Cancer-associated adipocytes are a powerful allay to proliferating cancer cells stimulating progression, invasion, and metastasis by secreting growth factors, adipokines, and proinflammatory cytokines [37]. Recently, a concept of cancer-adipose tissue metabolic symbiosis was introduced to describe nutritional and metabolic support provided by adipocytes through the supply of substrates for carbohydrate and lipid metabolism [20]. We analyzed tissue lactate concentration, LDH activity, and isozyme pattern, and LDHA/LDHB protein expression and localization in cancer-associated adipose tissue, compared to the breast adipose tissue of women with benign breast changes. We report, for the first time, depot-specific basal lactate metabolism in human breast adipose tissue and profound malignancy-associated changes to the lactate metabolism in cancer-associated adipose tissue. Namely, the results show significantly higher total LDH activity and provide evidence for the “harlequin phenomenon” in terms of non-homogenous protein localization of LDHB in cancer-associated adipose tissue.

High total LDH activity can be interpreted as an increased need for lactate production as well as lactate oxidation. In support of the former, adipose tissue is now recognized as one of the major body sites for lactate production [22,23], and several in vitro studies showed that lactate secreted from stromal cells contributes to cancer progression [38]. However, our results support the latter explanation, i.e., lactate oxidation by cancer-associated adipose tissue. Three primary LDH isozymes were found in adipose tissue, LDH3, and two predominantly “heart-type” isozymes, LDH2 and LDH1. Next to total LDH activity, the corresponding band intensity for these isozymes was stronger in cancer-associated adipose tissue than benign controls, while tissue lactate concentration did not differ. Moreover, immunohistochemical analysis showed that LDHB was primarily localized in cancer-associated adipocytes, especially where adipocytes are in direct contact with cancer cells. The ability of adipocytes to oxidize lactate is not surprising since adipocytes are highly equipped to respond to increased lactate stimulation from the tumor microenvironment through hydroxycarboxylic acid receptor 1 (GPR81) [39] as well as through monocarboxylate transporter (MCT) facilitated influx [40]. In lactate rich environments, adipocytes convert lactate to pyruvate to maintain cellular redox homeostasis and mitochondrial function [41]. Moreover, in conditions where adipocytes are competing for glucose with proliferating cancer cells, lactate oxidation can become a valuable resource for the maintenance of the acetyl-CoA pool and glyceroneogenesis. In light of the cell-specific protein localization of LDHB in adipocytes and LDHA in cancer cells, which was particularly prominent at the invasive front of the tumor, it is worth considering lactate oxidation by adipose tissue as part of the metabolic coupling in premenopausal breast cancer.

Furthermore, we hypothesized that systemic or local effects of obesity might bring forth changes in breast cancer lactate metabolism due to altered whole-body energy homeostasis or alterations in adipose tissue physiology, which could serve to explain differences in disease outcome in normal-weight and obese premenopausal women with breast cancer. Indeed, lactate concentration in malignant tumor tissue was significantly higher in normal-weight premenopausal women compared to overweight/obese premenopausal women with breast cancer. This staggering difference could not be attributed to changes in LDH activity, LDH isozyme pattern, or LDHA and LDHB protein expression. Similarly, LDH activity in cancer-associated adipose tissue was significantly higher in normal-weight women compared to overweight/obese women.

In conclusion, this study offers a contribution to the understanding of in vivo lactate metabolism in the context of malignancy and obesity in premenopausal women, highlighting the importance of lactate metabolism and its complexity within human breast cancer. Moreover, it sheds light on the metabolic reprogramming of cancer-associated adipose tissue, possibly indicating cancer-adipose tissue lactate-based metabolic coupling and opening the question of the existence of cancer-adipose lactate shuttle in premenopausal breast cancer. Finally, obesity-related differences in lactate concentration and LDH activity in cancer and cancer-associated adipose tissue, respectively, could have vast implications in terms of obesity-related differences in cancer aggressiveness, disease prognosis, and personalized therapeutic approaches. Further research is necessary to assess the potential of adipose tissue lactate metabolism to serve as a biomarker in terms of cancer progression, disease outcome, or therapy. To that end, our future studies will be directed towards in vitro and in vivo studies of lactate transport, signaling, and mitochondrial metabolism, as well as studies on tissue structure and cell ultrastructure in terms of LDH protein localization in breast cancer and cancer-associated adipose tissue in different types of breast cancer, with different histological grades, and at different stages of progression and metastasis.

## 4. Materials and Methods

### 4.1. Patient Selection and Sample Collection

The Ethics Committee of the Clinical Center of Vojvodina approved all procedures following the standards set by the latest version of the Helsinki Declaration (reference number: 4/19/1-1486/2-13). All participants involved in this study are volunteers who signed an informed consent form. Thirty-six premenopausal women (with regular menses for the last six months) with benign or malignant breast tumors were included in the study, following pathohistological assessment. Benign breast tumors were classified as fibroadenomas, while malignant breast tumors were classified as grade 2 and grade 3 luminal type A (ER+/PR+/HER2−) invasive ductal carcinoma with no or minimal involvement of the axillary lymph nodes. No women presented with metastasis or had undergone cancer therapy. Patient characteristics and sample pathohistological parameters are given in Table 1. During surgical intervention for these conditions, samples of the tumor tissue and tumor-associated adipose tissue were obtained. Adipose tissue in close proximity to the tumor mass (outside of the invasive front) was regarded as tumor-associated adipose tissue. To allow for the comparison between different methods and facilitate the integration of the results, each tissue sample was split into three parts for western blot, enzyme activity, and immunohistochemistry analyses, respectively. One piece was snap-frozen in liquid nitrogen and stored at −80 °C until subsequent protein isolation by the TRI Reagent procedure (Thermo Fisher Scientific, Waltham, Massachusetts, United States) for protein expression analysis by western blot. The second piece was snap-frozen in liquid nitrogen and stored at −80 °C until subsequent homogenization in Heidolph DIAX 600 (Heidolph Instruments, Schwabach, Germany) at 4 °C in 0.25 M sucrose, 0.1 mM EDTA, and 50 mM Tris buffer, pH 7.4 for enzyme activity measurements. The third piece was immediately fixed and stored in 4% PFA until subsequent dehydration and preparation of immunohistochemistry analysis. According to body mass index (BMI), samples were classified into four groups (*n* = 9) where women with the BMI < 25 kg/m^2^ were considered to be normal-weight, and women with BMI ≥ 25 kg/m^2^ were considered to be overweight/obese: normal-weight with benign tumors overweight/obese with benign tumors, normal-weight with malignant tumors, and overweight/obese with malignant tumors.

### 4.2. Measurement of Tissue Lactate Content

Tissue lactate content was determined in freshly snap-frozen tumor and adipose tissue samples prepared at 4 °C in sucrose buffer (0.25 M sucrose, 0.1 mM EDTA, and 50 mM Tris buffer, pH 7.4). A commercial kit for L-lactic acid measurement (BioSystems S.A., Barcelona, Spain) for IL ILab 300 Plus Chemistry Analyzer (Diamond Diagnostics, Holliston, MA, USA) was used. Results obtained in absolute unit mg/dL were normalized and expressed as a concentration in µM per mg of protein (determined by Lowry protein assay) or 100 mg of wet tissue mass.

### 4.3. Measurement of Lactate Dehydrogenase Activity

The activity of lactate dehydrogenase (LDH) was determined using the slightly modified method by Amnador [42] and expressed in nM NADH min^−1^ mg^−1^ protein. Tumor and adipose tissue protein sample prepared in sucrose buffer (0.25 M sucrose, 0.1 mM EDTA and 50 mM Tris buffer, pH 7.4) was added to the reaction mixture (NAD+ 5.25*10^−8^ M NAD, L-lactic acid 7.75*10^−2^ M in 0.1 M Tris-HCl, pH 8.5) and immediately transferred to a quartz cuvette. The increase in NADH absorbance at 340 nm was measured for 3 min at 30 °C. At specified substrate concentration and reaction mixture pH, more than 95% of the maximal activity was measured for all five isozymes. Thus, this method is equivalent to the measurement of total LDH activity.

### 4.4. Assessment of Isozyme Pattern of Lactate Dehydrogenase

Lactate dehydrogenase isoform pattern was determined by electrophoresis. In total, 5 µg and 10 µg of protein were loaded for tumor and adipose tissue samples, respectively (Figure 1 shows two representative bands for each group cut from the same gel, for full gels, see Supplementary Information Figure S1). Heart and liver protein samples were used as positive controls for the determination of electrophoretic migration lengths for all five LDH isozymes. Electrophoresis was performed in non-reducing non-denaturing conditions in 1% agarose gel in cold running buffer at constant current 100 V for 2 h. LDH isozymes and activity were visualized in the gel after incubation in 115 mM sodium lactate, 3.75 mg/mL NAD+, 0.075 mg/mL phenazine methosulfate, 0.75 mg/mL, and nitro blue tetrazolium. The gels were incubated at 37 °C for 20–30 min in the dark. Gels were subsequently scanned, and densitometric analysis was performed using ImageJ software (National Institute of Health, Bethesda, MD, USA).

### 4.5. Western Blot

Western blot analysis was done following a previously described procedure [43]. Antibodies against lactate dehydrogenase monomers and beta-actin were purchased from Abcam (Cambridge, United Kingdom) and used in the following concentrations: LDHA (1 µg/mL; ab47010), LDHB (0.1 µg/mL; ab85319), and β-actin (0.5 µg/mL; ab8226). Prior to loading onto the gel, nine samples from each group were pooled by three to obtain three final samples per group (Figure 2 shows two representative bands for each group cut from the same blot, for full blots, see Supplementary Information Figure S2). ImageJ software (National Institute of Health, Bethesda, Maryland, United States) was used for densitometric analyses of immunoreactive bands. Original band density was calculated as the sum of pixel intensities within a band. Final band density was obtained as the ratio of dots per band for the target protein averaged against β-actin (gel loading control). Protein expression is expressed in arbitrary units (AU) from three independent experiments.

### 4.6. Immunohistochemistry

Standard immunolabeling procedure was conducted as previously described [44] using primary antibodies against LDHA (1 µg/mL; ab47010) and LDHB (0.1 µg/mL; ab85319) and the appropriate mix of secondary antibodies (all purchased from Abcam, Cambridge, UK). Briefly, antigen retrieval was performed in citrate buffer on 4% PFA fixed, paraffin-embedded, 5µm serial sections. Incubation with primary antibodies was performed overnight at 4 °C, followed by incubation with secondary antibodies for 2 h at room temperature. Immunodetection was performed using the Dako LSAB Universal Kit (Agilent Technologies, Santa Clara, CA, USA). The sections were counterstained with hematoxylin, mounted, and examined with a Leica DMLB microscope (Leica Microsystems, Wetzlar, Germany).

### 4.7. Statistics

GraphPad Prism software (Version 6.01, GraphPad Software, San Diego, CA, USA) was used for all statistical analyses. D’Agostino and Pearson’s omnibus normality test was used for the assessment of normality of distribution for all data sets. One-way two-tailed analysis of variance (ANOVA) was applied for within-group comparison of the data from molecular analysis. If the F test showed an overall difference, multiple comparison Tukey’s post hoc test was used to evaluate the significance of the among group differences. Statistical significance was accepted at *p* < 0.05.

## Figures and Tables

**Table 1:** A table showing age in years (Mean±SD) and body mass index (BMI) in kg/m^2^ (Mean ± SD) of premenopausal women included in the study as well as pathohistological parameters for benign and malignant breast tumors; ^a^—compared to overweight/obese with benign tumors (*p* < 0.01), ^b^—compared to normal-weight with benign tumors (*p* < 0.001), ^c^—compared to normal-weight with malignant tumors (*p* < 0.001), N/A—not applicable.

	Groups			
Patient Characteristics	Normal-Weight with Benign Tumors (*n* = 9)	Overweight/Obese with Benign Tumors (*n* = 9)	Normal-Weight with Malignant Tumors (*n* = 9)	Overweight/Obese with Malignant Tumors (*n* = 9)
Age (y)	37.29 ± 8.01	33.60 ± 9.40	39.83 ± 7.39	46.67 ± 3.67 ^a^
BMI (kg/m^2^)	20.36 ± 2.12	28.23 ± 1.49 ^b^	22.68 ± 1.17	30.6 ± 4.29 ^c^
**Pathohistological parameters**				
Histological type	fibroadenoma	fibroadenoma	invasive ductal carcinoma	invasive ductal carcinoma
Hormone receptor status	N/A	N/A	ER+/PR+/HER2-	ER+/PR+/HER2-
Molecular subtype	N/A	N/A	luminal type A	luminal type A
**Tumor size**				
≤2 cm	4	5	6	5
>2 cm	5	4	3	4
**Histological grade**				
Grade II	N/A	N/A	5	6
Grade III	N/A	N/A	4	3
**Involvement of axillary lymph nodes**				
No involvement	N/A	N/A	7	6
Involvement of 1–3 axillary lymph nodes	N/A	N/A	2	3

## Supplementary Materials

Supplementary materials can be found at https://www.mdpi.com/1422-0067/21/24/9676/s1. Figure S1. Images of whole gels from native electrophoresis and LDH zymography for tumor and adipose tissue. Each image is representative of three independent trials. Each well represents a sample from one patient, showing two representative samples for tumor tissue and three representative samples for adipose tissue per group (normal-weight women with benign tumor, overweight/obese women with benign tumor, normal-weight women with malignant tumor, and overweight/obese women with malignant tumor, respectively). Figure S2. Images of whole blots for LDHA, LDHB, and β-actin obtained from the same membrane for tumor and adipose tissue, respectively. Each image is representative of three independent trials, showing three representative bands for tumor tissue and adipose tissue per group (normal-weight women with benign tumor, overweight/obese women with benign tumor, normal-weight women with malignant tumor, and overweight/obese women with malignant tumor, respectively). Prior to loading, nine samples from each group were pooled by three to obtain three samples shown in blots. For each target protein, two exposition times are given.

## Abbreviations

BMI	body mass index
GPR81	hydroxycarboxylic acid receptor 1
LDH	lactate dehydrogenase
LDH1	lactate dehydrogenase tetramer consisting of four LDHB subunits, also known as “heart-type” LDH
LDH2	lactate dehydrogenase tetramer consisting of three LDHB subunits and one LDHA subunit
LDH3	lactate dehydrogenase tetramer consisting of two LDHB subunits and two LDHA subunits
LDH4	lactate dehydrogenase tetramer consisting of one LDHB subunit and three LDHA subunits
LDH5	lactate dehydrogenase tetramer consisting of four LDHA subunits, also known as “muscle-type” LDH
LDHA	L-lactate dehydrogenase A chain
LDHB	L-lactate dehydrogenase B chain
MCT	monocarboxylate transporter
