# Supplementary material for: Lactate Metabolism in Breast Cancer Microenvironment: Contribution Focused on Associated Adipose Tissue and Obesity
Source: Int J Mol Sci. 2020 Dec 18;21(24):9676. doi: 10.3390/ijms21249676 (PMC7766866; doi:10.3390/ijms21249676)

Assessment of isozyme pattern of lactate dehydrogenase

Supplementary Figure 1. Images of whole gels from native electrophoresis and LDH zymography for tumor and adipose tissue. Each image is representative of three independent trials. Each well represents a sample from one patient, showing two representative samples for tumor tissue and three representative samples for adipose tissue per group (normal-weight women with benign tumor, overweight/obese women with benign tumor, normal-weight women with malignant tumor, and overweight/obese women with malignant tumor, respectively).

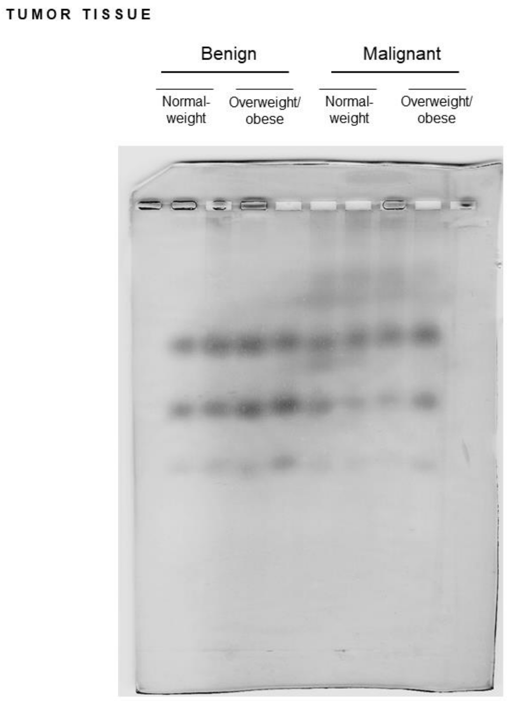

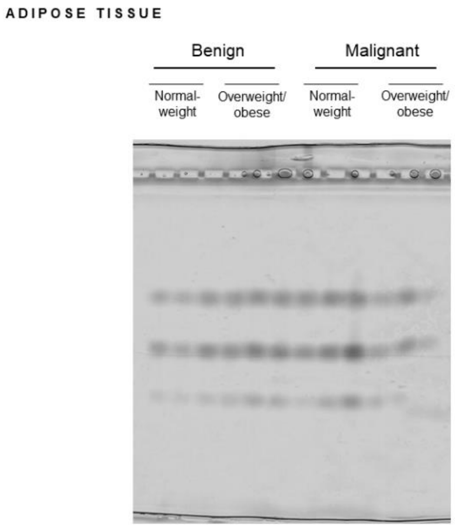

Western blot

Supplementary Figure 2. Images of whole blots for LDHA, LDHB, and β-actin obtained from the same membrane for tumor and adipose tissue, respectively. Each image is representative of three independent trials, showing three representative bands for tumor tissue and adipose tissue per group (normal-weight women with benign tumor, overweight/obese women with benign tumor, normal-weight women with malignant tumor, and overweight/obese women with malignant tumor, respectively). Prior to loading, nine samples from each group were pooled by three to obtain three samples shown in blots. For each target protein, two exposition times are given.

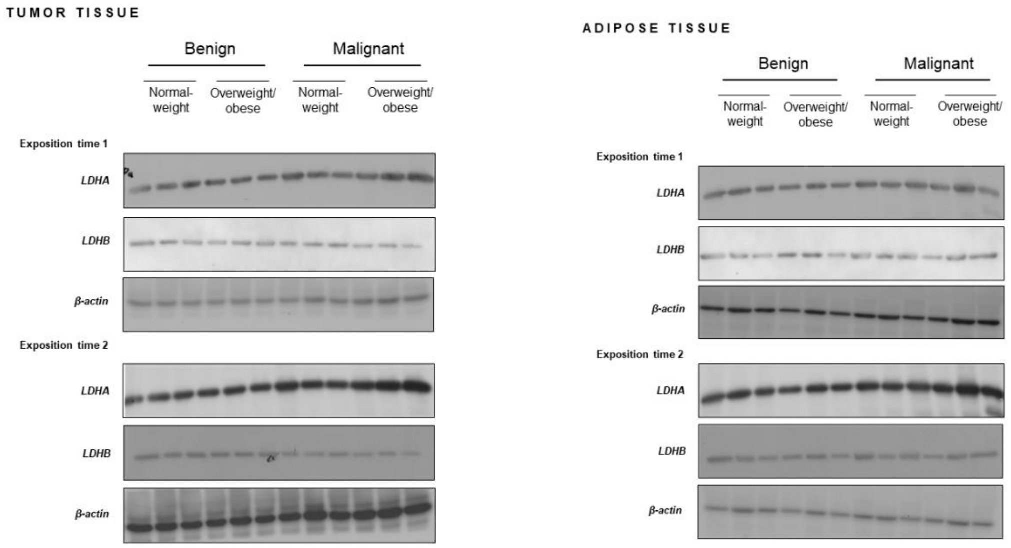

Supplement: Supplementary file 1 [file ijms-21-09676-s001.zip › Supplementary Information.pdf]
